# Stepping to the Beat: Feasibility and Potential Efficacy of a Home-Based Auditory-Cued Step Training Program in Chronic Stroke

**DOI:** 10.3389/fneur.2017.00412

**Published:** 2017-08-22

**Authors:** Rachel L. Wright, Simone Briony Brownless, David Pratt, Catherine M. Sackley, Alan M. Wing

**Affiliations:** ^1^School of Psychology, University of Birmingham, Birmingham, United Kingdom; ^2^School of Sport, Exercise and Rehabilitation Sciences, University of Birmingham, Birmingham, United Kingdom; ^3^West Midlands Rehabilitation Centre, Birmingham Community Healthcare Trust, Birmingham, United Kingdom; ^4^Faculty of Life Sciences and Medicine, King’s College London, London, United Kingdom

**Keywords:** locomotor training, hemiparesis, gait asymmetry, gait rehabilitation, stroke, auditory cueing

## Abstract

**Background:**

Hemiparesis after stroke typically results in a reduced walking speed, an asymmetrical gait pattern and a reduced ability to make gait adjustments. The purpose of this pilot study was to investigate the feasibility and preliminary efficacy of home-based training involving auditory cueing of stepping in place.

**Methods:**

Twelve community-dwelling participants with chronic hemiparesis completed two 3-week blocks of home-based stepping to music overlaid with an auditory metronome. Tempo of the metronome was increased 5% each week. One 3-week block used a regular metronome, whereas the other 3-week block had phase shift perturbations randomly inserted to cue stepping adjustments.

**Results:**

All participants reported that they enjoyed training, with 75% completing all training blocks. No adverse events were reported. Walking speed, Timed Up and Go (TUG) time and Dynamic Gait Index (DGI) scores (median [inter-quartile range]) significantly improved between baseline (speed = 0.61 [0.32, 0.85] m⋅s^−1^; TUG = 20.0 [16.0, 39.9] s; DGI = 14.5 [11.3, 15.8]) and post stepping training (speed = 0.76 [0.39, 1.03] m⋅s^−1^; TUG = 16.3 [13.3, 35.1] s; DGI = 16.0 [14.0, 19.0]) and was maintained at follow-up (speed = 0.75 [0.41, 1.03] m⋅s^−1^; TUG = 16.5 [12.9, 34.1] s; DGI = 16.5 [13.5, 19.8]).

**Conclusion:**

This pilot study suggests that auditory-cued stepping conducted at home was feasible and well-tolerated by participants post-stroke, with improvements in walking and functional mobility. No differences were detected between regular and phase-shift training with the metronome at each assessment point.

## Introduction

Stroke is a leading cause of long-term functional disability (1), with 70% of individuals post-stroke being classified as household or limited community ambulators based on their walking function (2, 3). Hemiparesis secondary to stroke typically results in a reduced walking speed and gait is generally asymmetrical with abnormal gait biomechanics (4). Gait asymmetry remains resistant to rehabilitation (5), and temporal asymmetry is present in over half of independent ambulatory stroke survivors (6). Large swing time asymmetries are associated with slower walking velocities (7) and with lower scores on the Berg Balance Scale (8), suggesting that this measure is an important marker for diminished balance. Rehabilitation of these gait features is a key goal for therapists as gait and balance are important for independent mobility and to reduce the risk of falling (9), as well as for quality of life (10).

The conventional view is that gait improves over the first 3–6 months following a stroke and then plateaus (11, 12). This view is widely used to discharge patients from motor rehabilitation programs after the initial acute phase (13). However, there is evidence that there are benefits of higher doses of training on outcome measures and that this is not influenced by time since stroke (14) (i.e., months or even years post-stroke). Furthermore, there is convincing evidence that gait training in the chronic phase can lead to continued improvement in walking speed (15, 16) and cardiovascular fitness (17, 18).

Auditory rhythm can produce an effect on the motor system, and studies have demonstrated the ability to synchronize lower limb movements to auditory cues (19, 20). Auditory cueing has been investigated as a means to improve hemiparetic gait. Chronic stroke participants are able to synchronize to a metronome during treadmill walking (21–23), and improvements in temporal symmetry were observed with acoustic pacing (21). Stepping in place to an auditory cue results in immediate reductions in both step time asymmetry and variability in participants with post-stroke hemiparesis consistent with those observed during cued walking (24). Auditory cueing has been used in stroke gait rehabilitation programs, with significantly greater improvements in walking speed and stride length in gait training with auditory cueing compared to conventional gait training (25) and Bobath training (26) and has been proposed as one of the most promising approaches to improving gait coordination (27).

Stepping in place is a skill that requires components found in gait, such as reciprocal flexion and extension of the lower limbs by timely coordination and synchronization to create a single limb support phase, a swing phase, and a step frequency (28). Therefore, this suggests stepping in place is an appropriate form of locomotor training for rehabilitation. The temporal asymmetries typically seen in hemiparetic walking are also shown during stepping in place (24, 28), suggesting that targeting asymmetry during stepping in place may have carry-over effects on everyday walking. If this is the case, it is possible that step training could be used to ensure high movement repetition where space is limited (e.g., in the home) as part of walking rehabilitation.

The ability to adapt walking to the demands of the environment is a key component of everyday mobility. Gait adaptability is reduced in people with stroke (22, 29, 30), which may contribute to the high incidence of falls during walking in stroke survivors (31, 32). Previous research involving correction to a phase shift in a metronome has shown that individuals with stroke delay correction if the perturbation occurs on the paretic limb (23) and that there is a preference for a slower step response when a faster step may be more optimal (22). The use of a variable metronome (e.g., with phase shifts) may help to simulate the temporal stepping adjustments encountered during community walking (i.e., curbs, uneven terrain). Therefore, repeated training of stepping adjustments may result in an improved ability to adapt gait during community walking.

The purpose of this pilot study was to investigate the feasibility and preliminary efficacy of home-based training involving auditory cueing with and without phase-shift adjustments of stepping in place. We hypothesized that the training would be tolerated by most individuals with minimal adverse events. In terms of efficacy of training, we formulated two research questions:

1. Does training of auditory cued stepping result in improved walking function?
2. Does training with auditory-cueing of stepping with phase-shift adjustments result in improved gait adaptability compared to standard auditory-cued step training?

We hypothesized that stepping training to an auditory metronome would lead to improvements in walking speed and functional mobility. We also hypothesized that training with phase-shift adjustments would improve scores on a measure of gait adaptability.

## Materials and Methods

### Participants

**Figure 1:**
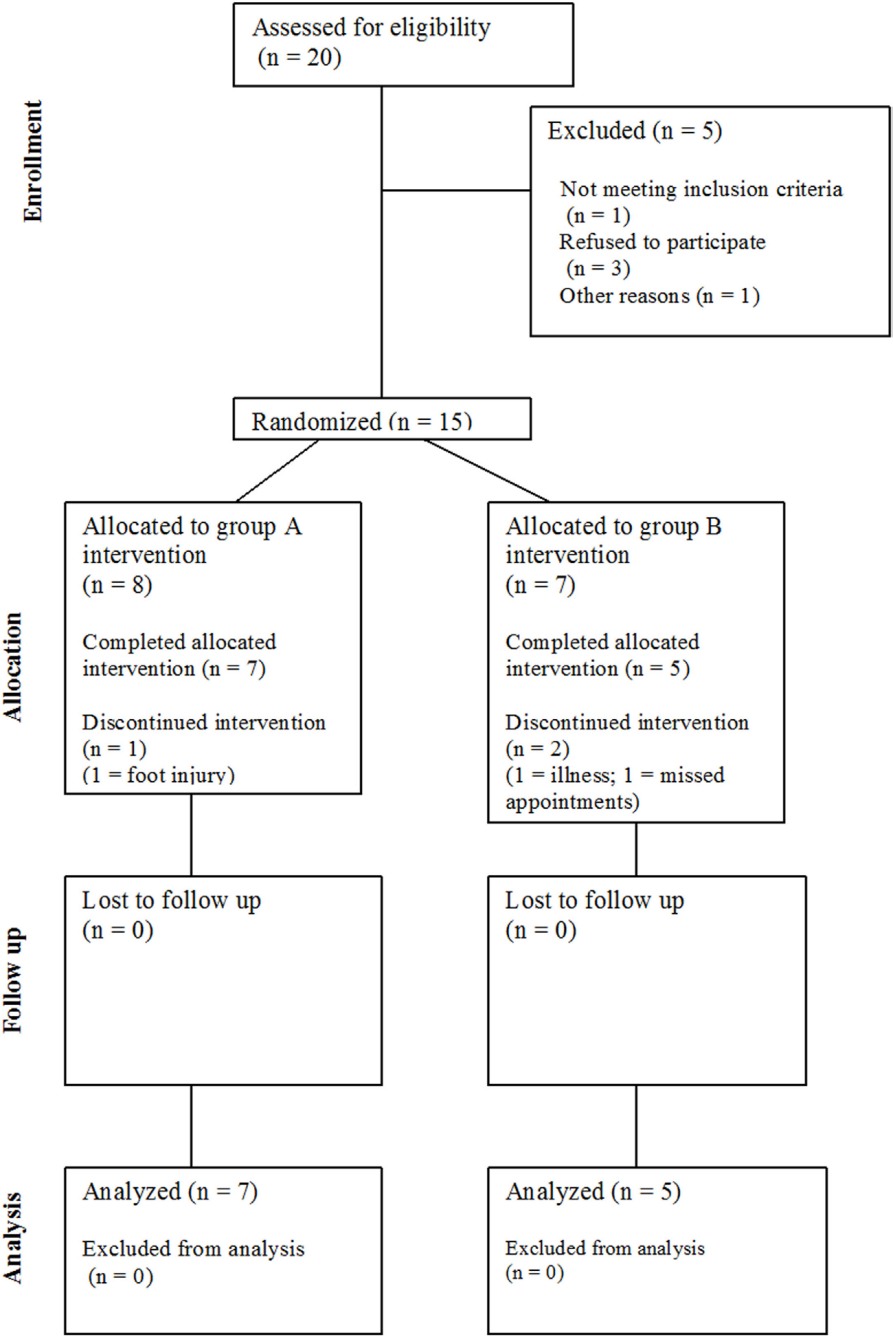
CONSORT diagram showing the flow of participants through the study.

Twenty community-dwelling stroke survivors were identified (see Figure 1), of which 15 eligible participants (four female) with post-stroke hemiparesis gave written, fully informed consent for this study. Favorable ethical opinion was granted by the South Birmingham Research Ethics Committee and was carried out in accordance with the principles laid down by the Declaration of Helsinki. Inclusion criteria were as follows: at least 6 months post-stroke, aged 18 years and over, walking disability but retained ability to stand and transfer, and a Rivermead Motor Assessment Gross Function (RMA GF) scale score (33) of 6–12 (maximum score 13). Participants were excluded if they had cognitive impairments preventing understanding of the task, insufficient command of English, or hearing impairments reducing ability to hear the metronome. Participant characteristics, such as demographics, time since stroke, affected side, walking aid usage, and fear of falling (34) were documented at baseline.

### Study Design

We used a repeated measures within-subject study cross-over design. The choice of design was based on the advantage of providing all treatment to all participants and increasing the statistical power within the constraints of research funding for this pilot study. Participants were randomly assigned to a block starting with standard metronome cue-training followed by phase-shifted metronome cue-training (Group A) or starting with phase-shifted metronome cue-training followed by standard metronome cue-training (Group B). Between the two training blocks, there was a 3-week wash-out period. After completion of the second training block, there was a final 3-week follow-up period to assess whether there were any carryover effects.

### Intervention

**Figure 2:**
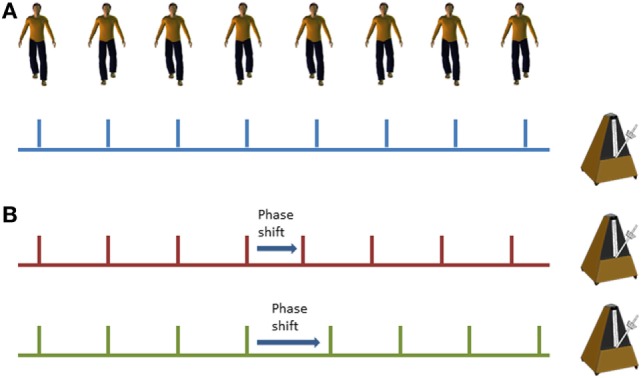
Illustration of standard metronome cues **(A)** and phase shifts within the metronome cues **(B)**; red line demonstrates a phase advance (shorter interval) and the green line demonstrates a phase delay (longer interval).

Cueing training was delivered in the home *via* mp3 player with either speakers or headphones (according to participant preference) set on a shuffle mode to vary the order of music presentation. Popular music in digital MIDI format was used to ensure temporal stability and precision. A metronome beat was overlaid to provide a clear rhythmical beat. For the phase shift condition, phase shifts of ±20% were inserted randomly into the music tracks at approximately 45–70 s intervals (see Figure 2). Cues were delivered initially at participant’s preferred natural stepping cadence (determined in the baseline assessment session) and were then increased by 5% on each subsequent training week by a researcher. On the first home visit, the researcher delivered the first training session to check suitability of the cueing tempo and to provide guidance if required to the participant. The participants were instructed to step in time to the metronome beat. Training was self-administered for 15 min on 5 days of the week in two 3-week blocks. Participants were informed that they could complete the training in multiple blocks (e.g., 3× 5 min sessions) through the day if fatigue meant 15 min was too much in one session. Participants received a weekly home visit from a researcher to encourage adherence to training, to facilitate the increase in speed of the prescribed metronome cue, and to address any concerns or questions that may have arisen. The research team were also contactable by telephone and email between the home visits in case any questions arose.

### Outcome Measures

The feasibility of the study was assessed by examining adherence, retention, and safety. A training diary was left at the participant’s home on during the training period so they could document when and for how long they trained for. It was stressed by the visiting researcher how important it was for the study that this was accurate and that any exaggeration on training duration could affect the study findings. Any adverse events (e.g., falls) or complaints of pain or fatigue were documented.

To establish if the research protocol was successful, some objective criteria were established to determine feasibility. Attrition was set at ≥15% based on the acceptable drop-out rate established by the PEDro scale (35). Adherence was set at ≥70% based on previous studies using adults with a physical impairment (36, 37). The intervention was considered safe if there were no falls or injuries associated with the stepping training.

As this study aimed to measure the training effects and not the immediate effects of cueing, efficacy outcome measures were tested without an auditory cue. The primary outcome measures to assess whether stepping training was beneficial were self-selected comfortable walking speed over 10-m and the 3-m Timed Up and Go (TUG) test (38) as a measure of functional mobility. Objective, quantitative data were obtained using Mobility Lab and six body-worn wireless Opal inertial measurement sensors (APDM Inc., Portland, OR, USA) for these measures, which have been shown to be valid and reliable for overground walking (39) and TUG measures (40). Opal units were worn on the sternum, waist, shanks, and feet according to the placement for gait and turning analysis. IWALK and GaitTrack within Mobility Lab software (APDM Inc., Portland, OR, USA) were used to obtain data for walking speed, step time, and swing time. The software automatically removes the first and last strides from walking trials to give steady-state walking speeds (39). Temporal asymmetry measures were calculated as a secondary outcome. Step time and swing time asymmetries were quantified using a ratio where the paretic time was divided by the non-paretic time (41). A value of 1 indicated symmetry between the paretic and non-paretic times, with values larger than 1 indicating a longer paretic than non-paretic time (i.e., the further the value away from 1, the higher the asymmetry). ITUG software (APDM Inc., Portland, OR, USA) was used to obtain TUG times (40).

The primary outcome measure to assess whether step training with phase shifts provided any additional benefits over standard training was the Dynamic Gait Index (DGI) (42). The DGI is an evaluation of an individual’s ability to modify gait in response to eight different task demands, e.g., stepping over an obstacle, and is the most extensively tested measure of walking adaptability in stroke populations (30). The DGI is scored out of 24, with higher scores indicating higher levels of mobility.

All efficacy outcome measures were assessed immediately prior to the first training block (baseline), after the first 3-week training block (Assessment 2), after the first 3-week break (Assessment 3), after the second 3-week training block (Assessment 4), and 3 weeks after the end of training (follow-up). All outcome measures were studied by the same un-blinded assessor.

### Statistical Analysis

Statistical analyses were conducted using SPSS version 21.0 (IBM Corp., Chicago, IL, USA). As the data were not normally distributed, non-parametric statistics were employed. To determine whether stepping training at home with auditory cues was beneficial, a related-samples Friedman’s Analysis of Variance was used to assess whether there were any differences between baseline (Assessment 1), post-training (Assessment 4), and follow-up (Assessment 5), using all participant data. If differences between assessments were detected, *post hoc* analysis using related-samples Wilcoxon Signed-Rank test with Bonferonni correction determined the location of these differences [i.e., baseline (Assessment 1) vs Assessment 4, baseline vs follow-up (Assessment 5) and Assessment 4 vs follow-up]. If a significant difference was observed, the effect size *r* was estimated from the *z*-score. To compare training with standard with phase-shift metronome training, independent Mann–Whitney U tests with Bonferroni adjustment were computed to determine training group differences at each assessment point for the walking speed, TUG time and DGI outcome variables. A significance level of *p* < 0.05 was used in all tests.

Data were also compared to published minimal detectable change values for walking speed (43), TUG time (44), DGI (45), and asymmetry ratios (46) to assess whether any observed changes would be considered clinically meaningful. Walking speed was also considered in terms of functional walking categories (2).

## Results

### Feasibility

Of 15 participants recruited to the study, data were excluded for 3 participants (20%) who did not complete the study; therefore, the attrition rate was higher than the 15% threshold. One participant withdrew due to illness, one participant withdrew due to a foot injury (unrelated to training), and a third participant was withdrawn by the research team after failing to be at home on several pre-arranged home visits. Therefore, 12 participants (80%) completed the intervention (see Table 1). There were no adverse events, e.g., falls, reported during training.

**Table 1:** Demographic and clinical features of the participants (*n* = 12; Mean ± SD unless stated).

Age (years)	56 ± 11
Sex	9 males; 3 females
Time since lesion (months)	68 ± 42
Affected side	7 left; 5 right
RMA GF (max 13)	10 ± 1
FES-I (max 64)	29 ± 7
Walking aid	9 yes; 3 no

*RMA GF, Rivermead Motor Assessment Gross Function; FES-I = Falls Efficacy Scale International*.

Based on the self-reported training diaries, 9 of the 12 participants (75%) completed all training sessions (i.e., 5× 15 min per week on all 3 weeks of both blocks) and, therefore, satisfied the criteria for adherence to training. Of the three participants who did not complete all training sessions: one participant completed 2 sessions of 10 min rather than 15 during the intervention, one participant missed one session of training but completed all other sessions, and one participant tended to complete 10–12 min of training each day rather than the full 15 min. Adherence was similar across training blocks A and B, but two participants stated they did not see the benefit of the phase shifts. Some participants suggested that increases and decreases in the tempo (i.e., speed) of the metronome/music within a training block might be useful. Overall, the participants reported that they enjoyed training, and five participants requested to keep the music once the study was completed.

### Efficacy of Stepping Training

Significant differences between assessments were detected for walking speed χ^2^(2) = 8.667, *p* = 0.013. Wilcoxon tests were used to follow up this finding with a Bonferroni-adjusted α-value of 0.017. Walking speed at assessments 4 (*p* = 0.005, *r* = −0.82) and 5 (*p* = 0.006, *r* = −0.79) were significantly quicker than at Assessment 1 (baseline). There was no difference in walking speed between Assessments 4 and 5 (*p* = 0.272), indicating that walking speed improvements were maintained at follow-up (see Table 2 for full results). The increases in walking speed exceeded the MDC value of 0.10 m⋅s^−1^ indicating this was a clinically meaningful improvement in walking speed. By follow-up, one participant had changed functional walking category from household to limited community ambulatory and two participants had changed from limited community to full community ambulatory.

**Table 2:** Outcome measures across the assessments (Median [IQR]).

		Assessment 1	Assessment 2	Assessment 3	Assessment 4	Assessment 5	χ^2^	*p*
Walking speed (m⋅s^−1^)	All (*n* = 12)	0.61 [0.32, 0.85]	0.73 [0.36, 0.94]	0.73 [0.38, 0.98]	0.76 [0.39, 1.03]*	0.75 [0.41, 1.03]	8.667	0.013
	Group A (*n* = 7)	0.66 [0.25, 0.87]	0.73 [0.24, 0.97]	0.73 [0.25, 0.99]	0.77 [0.22, 1.04]	0.76 [0.25, 1.04]		
	Group B (*n* = 5)	0.54 [0.35, 0.76]	0.72 [0.39, 0.99]	0.74 [0.40, 1.03]	0.76 [0.41, 1.06]	0.75 [0.42, 1.07]		

TUG (s)	All (*n* = 12)	20.0 [16.0, 39.9]	19.0 [14.6, 35.7]	17.0 [14.5, 36.6]	16.3 [13.3, 35.1]*	16.5 [12.9, 34.1]	18.500	<0.001
	Group A (*n* = 7)	19.8 [16.0, 53.7]	19.6 [15.8, 46.3]	17.1 [14.8, 45.3]	17.0 [14.3, 46.9]	15.7 [13.0, 42.2]		
	Group B (*n* = 5)	20.7 [12.7, 36.7]	18.4 [12.3, 33.4]	16.9 [12.2, 34.4]	15.6 [10.3, 33.3]	17.3 [10.1, 31.72]		

DGI (score; max 24)	All (*n* = 12)	14.5 [11.3, 15.8]	16.0 [12.3, 18.5]	14.5 [12.3, 18.0]	16.0 [14.0, 19.0]*	16.5 [13.5, 19.8]	10.364	0.006
	Group A (*n* = 7)	12.0 [11.0, 16.0]	16.0 [11.0, 16.0]	14.0 [11.0, 19.0]	16.0 [11.0, 20.0]	17.0 [11.0, 20.0]		
	Group B (*n* = 5)	15.0 [13.0, 17.0]	15.0 [12.5, 20.0]	15.0 [12.5, 17.5]	16.0 [14.0, 19.0]	16.0 [14.0, 19.5]		

Step Time Asymmetry (*n* = 9)		1.24 [1.14, 1.83]	1.21 [1.11, 1.42]	1.25 [1.14, 1.48]	1.22 [1.02, 1.32]	1.24 [1.08, 1.40]		

Swing Time Asymmetry (*n* = 9)		1.57 [1.19, 1.60]	1.40 [1.17, 1.66]	1.43 [1.18, 1.56]	1.32 [1.12, 1.50]	1.30 [1.19, 1.53]		

*Indicates a significant difference from baseline.

*TUG, Timed Up and Go; DGI, Dynamic Gait Index*.

Significant differences between assessments were detected for TUG time χ^2^(2) = 18.500, *p* < 0.001. Wilcoxon tests were used to follow up this finding, with a Bonferroni-adjusted α-value of 0.017. TUG times at assessments 4 (*p* = 0.004, *r* = −0.84) and 5 (*p* = 0.002, *r* = −0.88) were significantly shorter than those at baseline. Due to the Bonferonni-adjusted α-value, there was not a significant difference between assessments 4 and 5 (*p* = 0.028), suggesting that the training improvements are maintained at follow-up. Significant differences between assessments were also detected for DGI χ^2^(2) = 10.364, *p* = 0.006. Wilcoxon tests were used to follow up this finding, with a Bonferroni-adjusted *p*-value of 0.017. DGI scores at assessments 4 (*p* = 0.012, *r* = −0.72) and 5 (*p* = 0.010, *r* = −0.74) were significantly improved from baseline. There were no differences in scores between Assessments 4 and 5 (*p* = 0.100). Improvements on both TUG and DGI had exceeded MDC values post-stepping training. Therefore, these results indicate that stepping training results in improvements in functional mobility.

Due to technical issues with the Opal sensors, individual step and swing time measures for the walking trials were not available for three participants on one assessment. Therefore, there are only step and swing time ratio data for nine participants across all assessments and no statistics were run on these data due to the small remaining sample size. Visual inspection of the data suggests that the median step time symmetry ratio did not change over the course of the training (Table 2), whereas the median swing time asymmetry ratio appears to decrease at every assessment and exceeded the MDC value of 0.26 by follow-up.

### Efficacy of Step Training with Phase-Shifts

The primary outcome measure for assessing the efficacy of step training with phase-shifts was the DGI to assess gait adaptability. The independent Mann–Whitney U tests showed that there are no significant differences between treatment groups for DGI scores across the assessments [Assessment 1 (Baseline) *p* = 0.432, Assessment 2 *p* = 1.000, Assessment 3 *p* = 0.876, Assessment 4 *p* = 0.876, and Assessment 5 (Follow-up) *p* = 1.000]. Therefore, the hypothesis that training with phase-shifts would lead to greater improvements in gait adaptability than standard training was not supported.

There were also no significant differences between groups for walking speed across the assessments [Assessment 1 (baseline) *p* = 0.755, Assessment 2 *p* = 0.876, Assessment 3 *p* = 0.755, Assessment 4 *p* = 0.876, and Assessment 5 (Follow-up) *p* = 0.876]; or between groups for TUG time across the assessments [Assessment 1 (baseline) *p* = 0.755, Assessment 2 *p* = 0.639, Assessment 3 *p* = 0.530, Assessment 4 *p* = 0.530, and Assessment 5 (Follow-up) *p* = 0.639]. These results indicate the two groups were appropriately matched at baseline and that the improvements in these measures observed with stepping training were comparable regardless of whether the step training cueing was *via* a standard metronome or incorporated phase shifts.

## Discussion

This study assessed the feasibility and preliminary efficacy of home-based auditory cued stepping training in participants with post-stroke hemiparesis. Training was cued by metronome-enhanced music in two blocks with and without phase shifts for step adaptability training. Of those enrolled, 20% (three participants) dropped out or were removed during the first training block. Although attrition was higher than the 15% threshold, no participant withdrew from the study citing a lack of enjoyment in the intervention. There were no adverse events reported in relation to training. Our findings indicate the auditory-cued stepping training can be feasibly and acceptably delivered in the home, as there was a good adherence to the target treatment frequency. The results did not detect differences between standard and phase-shift training at each assessment point, but the training overall produced significant improvements in walking speed, TUG performance, and DGI performance. There was also a trend for a reduction in swing time asymmetry.

Increasing walking speed is a common goal in post-stroke rehabilitation for both therapists and stroke survivors. There was a significant increase in walking speed between baseline and post-step training. This increase for the median value was above the 0.10 m⋅s^−1^ considered a substantial meaningful change for the 10-min walk test (43), and visual inspection of the data revealed that this MDC value was achieved by Assessment 2 (after 3 weeks of training; Table 2). By considering functional walking categories based on walking speed (2), one participant had changed from household ambulatory to limited community ambulatory and two participants had changed from limited community to full community ambulatory by the end of the study. Transitioning to a higher category of ambulation is associated with a higher level of function, quality of life, and increased community participation (10).

The increase in walking speed was matched by improvements on functional mobility tests. A statistically significant improvement in TUG time occurred between baseline and post-stepping training. TUG time had improved more than the minimum detectable change (MDC; >2.9 s) value for this test from baseline by Assessment 4 (44), indicating that this improvement is clinically meaningful but also suggesting that a longer period of step training (6 vs 3 weeks) is needed for TUG to exceed the MDC value than was needed for walking speed. Previous research has also suggested that the time-course for TUG to improve above the MDC, in response to training, is longer than for walking speed (47). The improvement in score on the DGI between baseline and follow-up was greater than the 1.9 MDC value for older adults (45) but below the 2.6 MDC value for chronic stroke (48). Of the 12 participants, 10 scored lower than 19 on the DGI at the start of the study, indicating that these participants would be considered at higher risk of falling (49), with two participants transitioning from below this fall risk threshold to above this threshold by Assessment 4 (and maintained at follow-up).

Swing time asymmetries showed a tendency to reduce after training and had decreased by 0.27 between baseline and follow-up exceeding the MDC value (0.26) for this measure (46). Despite asymmetries during walking being a common feature in post-stroke hemiparesis, many people are capable of walking in a symmetrical fashion (50, 51). In addition, chronic stroke survivors may underestimate the magnitude of their walking asymmetry (52); therefore, asymmetries are likely to be established walking patterns in those with chronic hemiparesis (51). The motor system is very sensitive to stimulation by the auditory system (53); therefore auditory cueing may help to re-establish more symmetrical gait patterns through perceptual-motor anchoring (21).

There were no differences in outcome measures based on auditory cueing with or without phase shift perturbations. There have only been a small number of studies that have investigated step training in variable contexts, and these suggest that training is well tolerated with noticeable walking improvements (18, 36). To date, it has not been demonstrated that variable stepping training has advantages over conventional stepping training. However, as gait adaptability is reduced in post-stroke hemiparesis (22, 29, 30), it is feasible that stepping training with a variable context has benefits for gait stability and function that are yet to be documented. It is also possible that the DGI is not a sensitive enough instrument to detect improvements in gait adaptability between two slightly different training protocols and that a different outcome measure is warranted to assess this aspect of gait. Recent research has developed an overground gait adaptability test that may be a more suitable outcome measure for training in this context (36, 54).

Although physical activity was not monitored during home training, 9 of the 12 (75%) participants reported a completion of 15 min of step training on 5 days of the week and, therefore, the adherence criteria for the study was achieved. The remaining three participants reported an occasional day where they did not complete 15 min of training (e.g., 10 min rather than 15). Based on the mean tempo of the group during the first week of training (72 beats⋅min^−1^), this is a mean of 1,080 steps per day (5,040 steps per week). Individuals in the chronic phase post-stroke perform notably reduced amounts of daily stepping than sedentary older adults, even in those receiving physiotherapy (55). This suggests that individuals post stroke are not performing enough repetitions for neuroplasticity benefits in locomotor training, and sedentary behavior is commonly reported in individuals after stroke (56, 57) and is associated with multiple adverse health outcomes (58). Low levels of physical activity may lead to the loss of functional benefits from rehabilitation and is associated with a decline in mobility status in chronic stroke (59). A previous study, utilizing intensive locomotor training similar to the amount of stepping in the current study, showed improvements in walking performance after discharge from rehabilitation for reaching a “recovery plateau” and this was likely to be related to the high dose of training (17). Therefore, any gait intervention that provides a high dose of training is not only beneficial for continued improvement in mobility in chronic hemiparesis but is likely to also provide additional health benefits through increasing activity. A larger, follow-on study should monitor activity during training and also incorporate a measure of physical fitness (e.g., the 6-min walk test) to assess whether there are physiological improvements with training.

Rehabilitation at home is associated with improved functional outcomes and lower utilization of health services (60). Home-based exercise also has the advantage of being accessible to all participants, where transport to facilities may present a barrier to participation to some individuals. Previous research has shown that unsupervised, home-based exercise can lead to significant physical gains (61). All 12 participants reported they enjoyed taking part in the current study, and 5 participants requested to keep the training music to continue the step training once they had completed the study. Future research should investigate the feasibility and efficacy of physiotherapist-prescribed stepping training using auditory cueing as regular home-exercise between therapy sessions.

The primary limitations of this preliminary study included a lack of a control group and un-blinded assessors. Given that both walking speed and TUG times were measured using an objective, accelerometer-based system, we believe that subjective rater influence was limited. It cannot be excluded that similar findings may be achieved with an intervention of an equal intensity, but of a different modality. The small sample size may also limit the generalizability of the findings. The present data and derived effect sizes now permit the calculation of sample sizes for future randomized controlled trials in similar populations.

In summary, a home-based stepping training program with metronome-enhanced music was tolerated by all the participants for the two 3-week periods of training. Walking speed significantly increased with stepping training and was maintained at follow-up. Time Up and Go times significantly decreased and Dynamic Gait Index scores improved with stepping training. These findings are promising with respect to improvement in functional walking ability in chronic post-stroke hemiparesis and warrant future research, preferably a randomized controlled trial contrasting auditory-cued stepping training with another training modality [e.g., a structured home exercise program (62) or virtual reality training (63)].

## Ethics Statement

This study was carried out in accordance with the recommendations of the South Birmingham Research Ethics Committee with written informed consent from all subjects. All subjects gave written informed consent in accordance with the Declaration of Helsinki. The protocol was approved by the South Birmingham Research Ethics Committee.

## Author Contributions

Conceived and designed the experiments: RW, DP, CS, and AW. Data collection and analysis: RW and SB. Wrote the manuscript: RW. Critical revision of manuscript: SB, DP, CS, and AW. All the authors read and approved the final manuscript.

## Conflict of Interest Statement

The authors declare that the research was conducted in the absence of any commercial or financial relationships that could be construed as a potential conflict of interest.
